# Seroprevalence of HIV, HTLV, CMV, HBV and rubella virus infections in pregnant adolescents who received care in the city of Belém, Pará, Northern Brazil

**DOI:** 10.1186/s12884-018-1753-x

**Published:** 2018-05-16

**Authors:** Aubaneide Batista Guerra, Leonardo Quintão Siravenha, Rogério Valois Laurentino, Rosimar Neris Martins Feitosa, Vânia Nakauth Azevedo, Antonio Carlos Rosário Vallinoto, Ricardo Ishak, Luiz Fernando Almeida Machado

**Affiliations:** 1 Reference Unit Specialized in Maternal-Child and Adolescent Care, Belém, Pará Brazil; 2 Virology Laboratory, Institute of Biological Sciences, Federal University of Pará, Augusto Correa 1, Guamá, 66, Belém, Pará 075-110 Brazil; 3 Biology of Infectious and Parasitic Agents Graduate Program, Federal University of Pará, Belém, Pará Brazil

**Keywords:** Pregnant adolescents, Brazil, HIV, HTLV, HBV, CMV, Rubella, Seroepidemiology

## Abstract

**Background:**

Prenatal tests are important for prevention of vertical transmission of various infectious agents. The objective of this study was to describe the prevalence of human immunodeficiency virus (HIV), human T-lymphotropic virus (HTLV), hepatitis B virus (HBV), cytomegalovirus (CMV), rubella virus and vaccination coverage against HBV in pregnant adolescents who received care in the city of Belém, Pará, Brazil.

**Methods:**

A cross-sectional study was performed with 324 pregnant adolescents from 2009 to 2010. After the interview and blood collection, the patients were screened for antibodies and/or antigens against HIV-1/2, HTLV-1/2, CMV, rubella virus and HBV. The epidemiological variables were demonstrated using descriptive statistics with the G, χ^2^ and Fisher exact tests.

**Results:**

The mean age of the participants was 15.8 years, and the majority (65.4%) had less than 6 years of education. The mean age at first intercourse was 14.4 years, and 60.8% reported having a partner aged between 12 and 14 years. The prevalence of HIV infection was 0.3%, and of HTLV infection was 0.6%. Regarding HBV, 0.6% of the participants had acute infection, 9.9% had a previous infection, 16.7% had vaccine immunity and 72.8% were susceptible to infection. The presence of anti-HBs was greater in adolescent between 12 and 14 years old (28.8%) while the anti-HBc was greater in adolescent between 15 and 18 years old (10.3%). Most of the adolescents presented the IgG antibody to CMV (96.3%) and rubella (92.3%). None of the participants had acute rubella infection, and 2.2% had anti-CMV IgM.

**Conclusions:**

This study is the first report of the seroepidemiology of infectious agents in a population of pregnant adolescents in the Northern region of Brazil. Most of the adolescents had low levels of education, were susceptible to HBV infection and had IgG antibodies to CMV and rubella virus. The prevalence of HBV, HIV and HTLV was similar to that reported in other regions of Brazil. However, the presence of these agents in this younger population reinforces the need for good prenatal follow-up and more comprehensive vaccination campaigns against HBV due to the large number of women susceptible to the virus.

## Background

Some infectious agents, including human immunodeficiency virus (HIV), human T-lymphotropic virus (HTLV), human cytomegalovirus (CMV), hepatitis B virus (HBV) and rubella virus, are of great importance in the context of pregnancy due to the possibility of transmission from the woman to the baby during the gestational period, at the time of delivery and through breastfeeding [1–4]. Therefore, knowledge of the serological statuses of women is fundamentally important for the provision of good prenatal care to ensure that the necessary measures are taken to avoid the vertical transmission of these agents.

In Brazil, 99,804 pregnant women were infected with HIV as of June 2016, of whom 7.4% were residents of the northern region. The state of Pará showed an increasing trend in HIV detection rates in pregnant women between 1995 and 2015 and reported 2980 cases by 2016. In Brazil, the majority of pregnant women living with HIV/AIDS are between 20 and 24 years of age, and only 15.5% are between 10 and 19 years of age [5].

Approximately 20 million people worldwide are estimated to be infected with HTLV-1 or HTLV-2 [6]. In Brazil, the prevalence rates of HTLV-1/HTLV-2 infections in pregnant women are generally low. The prevalence is approximately 0.1% in the state of Mato Grosso do Sul [7, 8], approximately 0.1% in the city of Botucatu, São Paulo [9, 10], approximately 0.66% in Rio de Janeiro [10], approximately 0.3% in the state of Maranhão and approximately 1.5% in Bahia, which is the area with the highest prevalence in the country [11]. In the northern region of Brazil, the prevalence of HTLV-1/HTLV-2 infection varies from 0% in the state of Amazonas [12] to 0.3% in the state of Pará [13].

The prevalence of anti-CMV IgG antibodies in pregnant women is high in Brazil (97.0%) [14] and various regions of the world (91.3–99.5%) [15, 16]. However, CMV infection is still one of the most common causes of congenital malformation [17]. In Brazil, the diagnosis of acute rubella infection in pregnant women is very low [7, 18] due to the efficacy of the vaccine [19] and periodic vaccination campaigns.

Vertical transmission of HBV is also an important public health problem, mainly due to the increased risk of chronification of infection in children, especially if the mother has the HBV e antigen (HBeAg) and HBV surface antigen (HBsAg) markers during pregnancy or at the time of delivery [20, 21]. Thus, knowledge of the serological status for the virus has great relevance for the clinical management of these women during the gestational period.

In Brazil, several studies have reported the prevalence rates of HIV, HTLV, CMV, HBV and rubella in general populations of pregnant women [12, 18, 22, 23]. However, epidemiological information about these agents is still scarce in Brazil. No data are available for pregnant adolescents in the northern region of Brazil. Thus, an evaluation of the seroprevalence of these pathogens in pregnant adolescents is important to assess their early circulation and the rate of vaccine coverage against rubella and HBV.

The aim of the present study was to describe the seroprevalence rates of HIV, HTLV, HBV, CMV and rubella virus infections and to determine the vaccination coverage against HBV in pregnant adolescents who received care in the city of Belém, capital of the state of Pará, Brazil.

## Methods

### Type of study and ethical aspects

The present study is descriptive, cross-sectional and observational. The study was approved by the Human Research Ethics Committee of the Instituto Evandro Chagas under number 13/2009. The participants and their legal guardians were informed about the study objectives. After the adolescents and their legal guardians agreed to participate in the research, they signed a consent form and then answered an epidemiological questionnaire via a confidential structured interview. The questionnaire contained questions regarding age, place of birth and current residence, years of education, school dropout, age at first intercourse, condom use and age of the partner. Additionally, the vaccination card of the pregnant adolescents was evaluated to determine whether they had received the HBV vaccine.

### Sample size

The sample size determination was based on the estimated prevalence of viral infections in the general population. An estimated prevalence of 0.35% for HIV, 90% for CMV, 10% for HBV and 0.85% for HTLV resulted in a minimum sample size of 301 pregnant women. The sample error (ε) assumed in the present calculation was 5%, and a test power of 80% was established.

### Study population

A total of 324 pregnant adolescents who underwent prenatal care at the Reference Unit Specialized in Maternal-Child and Adolescent Care (Unidade de Referência Especializada Materno-Infantil Adolescência - UREMIA) under the Executive Secretariat of Public Health of the State of Pará (Secretaria Executiva de Saúde Pública do Estado do Pará - SESPA) participated in the study from November 2009 to February 2010.

Adolescents ranging between 12 and 18 years of age at any stage of pregnancy and of any religion, ethnicity, and origin who agreed to participate in the study were included. Pregnant women under 12 and over 18 years of age were excluded, as were those who had known serology results for the agents studied or who did not agree to participate.

### Serology

A 10-mL aliquot of peripheral blood was collected from each participant and placed in a tube containing ethylenediaminetetraacetic acid (EDTA) as an anticoagulant. The samples were transported to the Virology Laboratory of the Biological Sciences Institute (Instituto de Ciências Biológicas - ICB), Federal University of Pará (Universidade Federal do Pará - UFPA). Plasma and formed elements were separated by centrifugation at 8944 g for 10 min, transferred to an Eppendorf tube and frozen at − 20 °C prior to the serological and molecular biology analyses.

Anti-HIV antibody screening was performed using the ELISA method (Tetra Elisa kit, Biotest AG, Dreiech, Germany). The reactive samples were confirmed using the microparticle enzyme immunoassay method (MEIA, Abbott Axsym™ System, HIV-1/2, Abbott GmbH, Weisbaden, Delkenheim, Germany), followed by confirmation by indirect immunofluorescence (Bio-Manguinhos Fiocruz, Brazil).

Screening for anti-HTLV-1/HTLV-2 antibodies was performed using an ELISA immunoenzymatic assay (HTLV-1 + 2 Ab-Captures ELISA Test System; Ortho Clinical Diagnostic Inc., New Jersey, USA). Reactive samples were confirmed using nested PCR as previously described [24].

Serology for the HBsAg, HBV surface antibodies (anti-HBs) and total anti-HBc markers was performed by ELISA (ETI-AB-AUK-3, DiaSorin, Saluggia, Italy and ETI-MAK-2. PLUS, Diasorin, Italy). Samples that showed reactivity to total anti-HBc were tested for the presence of anti-HBc IgM. The detection of IgM and IgG for CMV and rubella virus was performed by ELISA (DiaSorin, Saluggia, Italy).

### Statistical analysis

The Chi-square test (χ^2^), G-test and Fisher’s exact test were used to assess differences in proportions between the reactivity to the tested agents and the sociodemographic and epidemiological variables of the adolescents, including age, level of education, use of condoms during sexual intercourse and age of partner. Results with *p* < 0.05 were considered significant.

## Results

The Table 1 presents the characteristics of pregnant adolescents. The mean age of the pregnant adolescents was 15.8 years (range from 12 to 18 years). The majority was between 15 and 18 years of age, lived in the capital of the state of Pará, had less than 6 years of education and continued to study. Regarding the onset of sexual activity of the adolescents, the mean age at first intercourse was 14.4 years and the mean age of the partners at first intercourse was 19.8 years. The frequency of male condom use in sexual intercourse is low and few participants were previously vaccinated against HBV according vaccination cards of the adolescents.

**Table 1:** Sociodemographic characteristics of the group of pregnant adolescents who received care in Belém, Pará, from November 2009 to February 2010

Demographic variable	Number	Percent
Age group		
12 to 14	52	16.1
15 to 18	272	83.9
Residence		
Capital	248	76.5
Interior	76	23.5
Years of education		
< 6	212	65.4
6–9	102	31.5
> 9	10	3.1
School dropout		
Yes	107	33.0
No	186	57.4
Did not attend school	31	9.6
Age at first intercourse		
9 to 11	6	1.9
12 to 14	197	60.8
15 to 17	121	37.3
Age of partner		
12 to 18	150	46.3
19 to 25	146	45.0
26 to 42	28	8.7
Condom use		
Sometimes	249	76.9
Never	75	23.1
Gestational age		
1 to 2 months	104	32.1
3 to 4 months	98	30.2
5 to 6 months	112	34.6
7 to 9 months	10	3.1
Vaccination against HBV		
No	228	70.4
Yes	87	26.8
Unknown	9	2.8

The prevalence of HIV-1 infection was 0.3% (1/324). This adolescent was 17 years old, from the city of Belém, had 6 years of education and reported that she had dropped out of school when she found out about the pregnancy. Her first sexual intercourse was at age 14 without the use of a male condom and was with a 20-year-old sexual partner; both were users of non-injectable drugs. Regarding sexual practices, the adolescent reported a daily frequency of sexual intercourse and sporadic anal sex. She mentioned having had two sexual partners, one of whom was a non-injectable drug user from another state in Brazil. For HTLV, the prevalence of infection was 0.6% (2/324). These two adolescents were 15 and 16 years of age and in the first trimester of their first pregnancy. The younger woman resided in the municipality of Gurupá in the interior of the state of Pará, was married and had 8 years of education, whereas the other was single, had 6 years of education and resided in the city of Belém, also in Pará.

Regarding hepatitis B infection, 0.6% (2/324) of the adolescents presented recent infection (HBsAg and anti-HBc IgM positive), and only 16.7% (54/324) showed vaccine immunity to HBV (presence of isolated anti-HBs). A history of previous HBV infection was observed in 9.9% (32/324) of the adolescents (presence of total anti-HBc and anti-HBs); however, most of the adolescents (72.8%, 236/324) presented a serological profile of susceptibility to the virus. This finding was in agreement with the observations from the vaccination cards of the adolescents, which indicated that 70.4% of the participants were not previously vaccinated.

The statistical analysis showed no differences in proportions between the participants who were reactive and nonreactive for total anti-HBc according to the age group (Fisher’s exact test; *p* = 0.56); however, we observed a difference in the proportions between the groups according to the age of the sexual partner (G test, *p* = 0.01) and the use of condoms in sexual intercourse (*p* = 0.01). We also observed that the age of the adolescents (χ^2^, *p* = 0.01), the level of education (G test, *p* = 0.02) and the age of the sexual partner (G test, *p* = 0.02) were factors that influenced the proportion of adolescents who were positive and negative for anti-HBs (Table 2).

**Table 2:** Epidemiological characteristics in association with the presence of serological markers of CMV infection, HBV infection and HBV vaccine immunity of pregnant adolescents who received care in Belém, Pará, from November 2009 to February 2010

Variables	N (%)	Anti-CMV IgM		*P* value	Anti-HBc Total		*P* value	Anti-HBs		*P* value
		Pos (%)	Neg (%)		Pos (%)	Neg (%)		Pos (%)	Neg (%)	
Age Group										
12–14	52 (16.0)	3 (5.8)	49 (94.2)	0.08^a^	4 (7.7)	48 (92.3)	0.56^a^	15 (28.8)	37 (71.2)	0.01^b^
15–18	272 (84.0)	4 (1.5)	268 (98.5)		28 (10.3)	244 (89.7)		39 (14.3)	233 (85.7)	
Years of education										
< 6	212 (65.4)	4 (2.0)	198 (98.0)		20 (9.4)	192 (90.6)		22 (10.4)	190 (89.6)	
6–9	102 (31.5)	2 (2.0)	100 (98.0)	0.44^c^	10 (9.8)	92 (90.2)	0.62^c^	29 (28.4)	73 (71.6)	0.02^c^
> 9	10 (3.1)	1 (10.0)	9 (90.0)		2 (20.0)	8 (80.0)		3 (30.0)	7 (70.0)	
Age at first intercourse										
9 to 11	6 (1.8)	0 (0.0)	6 (100.0)		1 (16.7)	5 (83.3)		2 (33.3)	4 (66.7)	
12 to 14	197 (60.8)	3 (1.5)	194 (98.5)	0.9^c^	19 (9.6)	178 (90.4)	0.88^c^	32 (16.2)	165 (83.8)	0.62^c^
15 to 17	121 (37.3)	4 (3.3)	117 (96.7)		12 (9.9)	109 (90.1)		20 (16.5)	101 (83.5)	
Condom use										
Sometimes	249 (76.9)	5 (2.0)	244 (98.0)	0.9^a^	13 (5.2)	236 (94.8)	0.01^a^	41 (16.5)	208 (83.5)	0.86^c^
Never	75 (23.1)	2 (2.7)	73 (97.3)		19 (25.3)	56 (74.7)		13 (17.3)	62 (82.7)	
Age of partner (years)										
12 to 18	150 (46.3)	4 (2.7)	146 (97.3)		9 (6.0)	141 (94.0)		25 (16.7)	125 (83.3)	
19 to 25	146 (45.0)	3 (2.0)	143 (98.0)	0.5^c^	12 (8.2)	134 (91.8)	0.01^c^	19 (13.0)	127 (87.0)	0.02^c^
25 to 42	28 (8.7)	0 (0.0)	28 (100.0)		11 (39.3)	17 (60.7)		10 (35.7)	18 (64.3)	

^a^Fisher’s exact test; ^b^Chi-square test; ^c^G test

Regarding CMV, 2.2% of the adolescents had anti-IgM positive, 96.3% had only IgG antibodies, and only 1.5% did not present either of the two antibodies. Most of the participants presented rubella IgG (92.3%), and no acute infection by this agent was observed. No significant difference in any of the categorical variables assessed in the study was observed in the proportions of individuals who tested positive and negative for anti-CMV IgM (Table 2).

## Discussion

The present study reported for the first time the serological profiles for HTLV, HIV, HBV, CMV and rubella in pregnant adolescents who received care in the city of Belém, Pará, northern region of Brazil. Although the prevalence of HTLV in the study population (0.6%) was within the range previously observed in other populations in the state of Pará [24, 25], we considered the prevalence high compared to other studies performed in pregnant women in the state of Pará [13] and other locations in Brazil, including Manaus [12], Maranhão [26], Paraná [18] and Maceió [23]. This finding is worrying because it indicates early contact of young women with an agent whose screening is not included in the prenatal tests and whose main forms of transmission include breastfeeding. Notably, one of the adolescents resided in the capital and another in the interior of the state of Pará, which indicated a need for greater epidemiological surveillance of this agent in prenatal services throughout the state.

A different scenario was observed for HIV-1, since the prevalence of infection in pregnant adolescents in Pará (0.3%) was similar to the prevalence found in pregnant women in the state of Amazonas [12]; this result confirmed a low incidence of infection in the northern region of Brazil. Our results are in agreement with data from the Ministry of Health that note a higher number of cases of HIV-1 infection in women between the ages of 20 and 24 years [5]. In Brazil, other studies in non-adolescent pregnant women demonstrated a low seroprevalence of HIV-1 infection [7, 23, 27, 28], which differed from reports from other developing countries, where the prevalence of infection was greater than 5% [29–31].

Regarding serology for HBV, almost 10% of the adolescents in the present study had a profile of virus infection. This early contact with the virus has also been observed in several regions of Brazil [32]. The prevalence of recent HBV infection (0.6%) was low in the study population; however, most adolescents (83.3%) presented a serological profile of susceptibility to HBV infection, with an absence of anti-HBV antibodies. This finding indicates the need for greater epidemiological surveillance for HBV in this population of pregnant women. This surveillance should aim to increase vaccination coverage to avoid neonatal or vertical infection by this viral agent, which is associated with a greater probability of chronification of infection and the development of hepatic cirrhosis and hepatocellular carcinoma in infected children.

A low prevalence of HBV in the population of pregnant women has also been observed in several other regions of Brazil [18, 32–34] and other countries [35]. These prevalence rates were much lower than the prevalence rates found in pregnant women in Asian [36, 37] and African countries [38, 39]. No case of coinfection between HIV, HTLV and HBV was observed in the adolescents participating in the present study. Additionally, we observed that the presence of anti-HBs might be related to the age of the adolescent, the level of education and the age of the sexual partner, and the presence of anti-HBc was influenced by the non-use of condoms in sexual intercourse and the partner’s age. In Brazil, few studies have associated epidemiological variables with serological markers of HBV infection other than HBsAg. In Maranhão, the presence of anti-HBc was associated with the level of education in pregnant women in general [40], but no association was observed in other Brazilian states, including Goiás [34] and Espírito Santo [41]. This finding indicates the need for better monitoring of these markers for the implementation of public policies to combat HBV. There are some limitations in this study specially in relation to hepatitis markers, such as anti-HBe, anti-HCV and anti-HDV which were not included in the present project due to lack of financial resources, however, in our future studies we will address these limitations.

Few studies have investigated the prevalence of CMV in the population of pregnant adolescents in Brazil. The present article is the first to report the high seroprevalence of anti-CMV IgG in pregnant adolescents from Pará (96.3%), which is similar to observations in pregnant women aged 12 to 19 years in Ribeirão Preto [14] and pregnant women in general from Mato Grosso do Sul [7], Espírito Santo [42] and developing countries [43, 44]. However, a higher percentage (2.2%) of pregnant adolescents in our study presented anti-CMV IgM (reactivation, reinfection or recent infection) compared to pregnant women from Mato Grosso do Sul (0.05%), where the age group was older [7]. This scenario is worrying, because CMV is one of the main infectious agents associated with congenital malformation. Thus, pregnancy at an early age carries a higher risk of infection and consequent vertical transmission of CMV.

No pregnant adolescent with acute rubella infection was detected, but 7.7% of the adolescents were susceptible to the virus, which reinforced the need for rubella vaccination campaigns to achieve greater coverage in Brazil and avoid the onset of congenital rubella syndrome. Similar results were reported in other regions of Brazil [7, 45, 46].

Our study had some limitations, such as the short time period for data collection and the impossibility of finding a statistical association between the presence or absence of infection with HIV or HTLV and acute infection with HBV or rubella virus based on epidemiological data, including the age, condom use and age at first intercourse, due to the low number of participants who presented positive serology for any of the evaluated markers. This shortcoming can most likely be overcome by increasing the sample size in future studies.

## Conclusions

In conclusion, the prevalence of HIV, HTLV and HBV infection was similar to the prevalence rates reported in other states of Brazil and showed early contact of adolescents with some agents of parenteral transmission. Acute rubella infection was not observed; however, the occurrence of CMV infection (recent or reativaction) was higher than reported in pregnant women in general in Brazil, demonstrating the importance of prenatal follow-up from the beginning of pregnancy to reduce the onset of congenital cytomegalovirus. Finally, we found that the majority of adolescents were susceptible to HBV infection, which reinforced the need for public policies aimed at intensifying the immunization of this specific population against HBV due to the high rate of infection chronicity in children infected at birth or in the first year of life.

## Abbreviations

anti-HBc: Antibody to hepatitis B core antigen
anti-HBe: Antibody to hepatitis B e antigen
anti-HBs: Antibody to hepatitis B surface antigen
anti-HCV: Antibody to hepatitis C virus
anti-HDV: Antibody to hepatitis D virus
CMV: Cytomegalovirus
EDTA: Ethylenediaminetetraacetic acid
ELISA: Enzyme-linked immunosorbent assay
HBeAg: Hepatitis B e antigen
HBsAg: Hepatitis B surface antigen
HBV: Hepatitis B virus
HIV: Human immunodeficiency virus
HTLV: Human T-lymphotropic virus
ICB: Biological Sciences Institute
PCR: Polymerase chain reaction
SESPA: Executive Secretariat of Public Health of the State of Pará
UFPA: Federal University of Pará
UREMIA: Reference Unit Specialized in Maternal-Child and Adolescent Care
χ^2^: Chi-square test

## References

[1] Margioula-Siarkou C, Kalogiannidis I, Petousis S, Prapa S, Dagklis T, Mamopoulos A (2015). Cytomegalovirus, toxoplasma gondii and rubella vertical transmission rates according to mid-trimester amniocentesis: a retrospective study. Int J Prev Med.

[2] Mendes MS, Costa MC, Costa IM (2016). Human T-cell lymphotropic virus-1 infection: three infected generations in the same family. Rev Soc Bras Med Trop.

[3] Kang W, Li Q, Shen L, Zhang L, Tian Z, Xu L (2017). Risk factors related to the failure of prevention of hepatitis B virus mother-to-child transmission in Yunnan, China. Vaccine.

[4] Nakamura KJ, Heath L, Sobrera ER, Wilkinson TA, Semrau K, Kankasa C (2017). Breast milk and in utero transmission of HIV-1 select for envelope variants with unique molecular signatures. Retrovirology.

[5] Brasil (2016). Boletim Epidemiológico AIDS e DST.

[6] Gessain A, Cassar O (2012). Epidemiological aspects and world distribution of HTLV-1 infection. Front Microbiol.

[7] Figueiró-Filho EA, Senefonte FR, Lopes AH, de Morais OO, Souza Júnior VG, Maia TL (2007). Frequency of HIV-1, rubella, syphilis, toxoplasmosis, cytomegalovirus, simple herpes virus, hepatitis B, hepatitis C, Chagas disease and HTLV I/II infection in pregnant women of state of Mato Grosso do Sul. Rev Soc Bras Med Trop.

[8] Dal Fabbro MM, Cunha RV, Bóia MN, Portela P, Botelho CA, Freitas GM, Soares J, Ferri J, Lupion J (2008). HTLV 1/2 infection: prenatal performance as a disease control strategy in state of Mato Grosso do Sul. Rev Soc Bras Med Trop.

[9] Olbrich Neto J, Meira DA (2004). Soroprevalence of HTLV-I/II, HIV, siphylis and toxoplasmosis among pregnant women seen at Botucatu - São Paulo - Brazil: risk factors for HTLV-I/II infection. Rev Soc Bras Med Trop.

[10] Monteiro DL, Taquette SR, Sodré Barmpas DB, Rodrigues NC, Teixeira SA, Villela LH, Bóia MN, Trajano AJ (2014). Prevalence of HTLV-1/2 in pregnant women living in the metropolitan area of Rio de Janeiro. PLoS Negl Trop Dis.

[11] Mello MA, da Conceição AF, Sousa SM, Alcântara LC, Marin LJ, Regina da Silva Raiol M, Boa-Sorte N, Santos LP, de Almeida Mda C, Galvão TC, Bastos RG, Lázaro N, Galvão-Castro B, Gadelha SR (2014). HTLV-1 in pregnant women from the southern Bahia, Brazil: a neglected condition despite the high prevalence. Virol J.

[12] Machado Filho AC, Sardinha JF, Ponte RL, Costa EP, da Silva SS, Martinez-Espinosa FE (2010). Prevalence of infection for HIV, HTLV, HBV and of syphilis and chlamydia in pregnant women in a tertiary health unit in the western Brazilian Amazon region. Rev Bras Ginecol Obstet.

[13] Sequeira CG, Tamegão-Lopes BP, Santos EJ, Ventura AM, Moraes-Pinto MI, Succi RC (2012). Descriptive study of HTLV infection in a population of pregnant women from the state of Pará, northern Brazil. Rev Soc Bras Med Trop.

[14] Yamamoto AY, Castellucci RA, Aragon DC, Mussi-Pinhata MM (2013). Early high CMV seroprevalence in pregnant women from a population with a high rate of congenital infection. Epidemiol Infect.

[15] El Sanousi SM, Osman ZA, Mohmed AB, Al Awfi MS (2016). Cytomegalovirus infection in a cohort of pregnant women. Am J Infect Control.

[16] Numan O, Vural F, Aka N, Alpay M, Coskun AD (2015). TORCH seroprevalence among patients attending obstetric Care Clinic of Haydarpasa Training and Research Hospital affiliated to Association of Istanbul Northern Anatolia Public Hospitals. North Clin Istanb.

[17] Mussi-Pinhata MM, Yamamoto AY, Moura Brito RM, de Lima Isaac M, de Carvalho e Oliveira PF, Boppana S (2009). Birth prevalence and natural history of congenital cytomegalovirus infection in a highly seroimmune population. Clin Infect Dis.

[18] Ferezin RI, Bertolini DA, Demarchi IG (2013). Prevalence of positive sorology for HIV, hepatitis B, toxoplasmosis and rubella in pregnant women from the northwestern region of the state of Paraná. Rev Bras Ginecol Obstet.

[19] Robertson SE, Featherstone DA, Gacic-Dobo M, Hersh BS (2003). Rubella and congenital rubella syndrome: global update. Rev Panam Salud Publica.

[20] Degli Esposti S, Shah D (2011). Hepatitis B in pregnancy: challenges and treatment. Gastroenterol Clin N Am.

[21] Piratvisuth T (2013). Optimal management of HBV infection during pregnancy. Liver Int.

[22] Maia MM, Lage EM, Moreira BC, Deus EA, Faria JG, Pinto JA, Melo VH (2015). Prevalence of congenital and perinatal infection in HIV positive pregnant in Belo Horizonte metropolitan region. Rev Bras Ginecol Obstet.

[23] Moura AA, de Mello MJ, Correia JB (2015). Prevalence of syphilis, human immunodeficiency virus, hepatitis B virus, and human T-lymphotropic virus infections and coinfections during prenatal screening in an urban northeastern Brazilian population. Int J Infect Dis.

[24] Vallinoto AC, Pontes GS, Muto NA, Lopes IG, Machado LF, Azevedo VN (2006). Identification of human T-cell lymphotropic virus infection in a semi-isolated afro-Brazilian Quilombo located in the Marajó Island (Pará, Brazil). Mem Inst Oswaldo Cruz.

[25] Santos EL, Tamegão-Lopes B, Machado LF, Ishak MO, Ishak R, Lemos JA (2009). Molecular characterization of HTLV-1/2 among blood donors in Belém, state of Pará: first description of HTLV-2b subtype in the Amazon region. Rev Soc Bras Med Trop.

[26] Guimarães de Souza V, Lobato Martins M, Carneiro-Proietti AB, Januário JN, Ladeira RV, Silva CM (2012). High prevalence of HTLV-1 and 2 viruses in pregnant women in São Luis, state of Maranhão, Brazil. Rev Soc Bras Med Trop.

[27] Domingues RM, Szwarcwald CL, Souza PR, Leal MC (2015). Prenatal testing and prevalence of HIV infection during pregnancy: data from the &quot;birth in Brazil&quot; study, a national hospital-based study. BMC Infect Dis.

[28] Lima KO, Salustiano DM, Cavalcanti AM, Leal ÉS, Lacerda HR (2015). HIV-1 incidence among people seeking voluntary counseling and testing centers, including pregnant women, in Pernambuco state, Northeast Brazil. Cad Saude Publica.

[29] Manyahi J, Jullu BS, Abuya MI, Juma J, Ndayongeje J, Kilama B (2015). Prevalence of HIV and syphilis infections among pregnant women attending antenatal clinics in Tanzania, 2011. BMC Public Health.

[30] Endris M, Deressa T, Belyhun Y, Moges F (2015). Seroprevalence of syphilis and human immunodeficiency virus infections among pregnant women who attend the University of Gondar teaching hospital, Northwest Ethiopia: a cross sectional study. BMC Infect Dis.

[31] Hokororo A, Kihunrwa A, Hoekstra P, Kalluvya SE, Changalucha JM, Fitzgerald DW (2015). High prevalence of sexually transmitted infections in pregnant adolescent girls in Tanzania: a multi-community cross-sectional study. Sex Transm Infect.

[32] Brasil (2015). Boletim Epidemiológico - Hepatites Virais.

[33] Boa-Sorte N, Purificação A, Amorim T, Assunção L, Reis A, Galvão-Castro B (2014). Dried blood spot testing for the antenatal screening of HTLV, HIV, syphilis, toxoplasmosis and hepatitis B and C: prevalence, accuracy and operational aspects. Braz J Infect Dis.

[34] Fernandes CN, Alves Mde M, de Souza ML, Machado GA, Couto G, Evangelista RA (2014). Prevalence of seropositivity for hepatitis B and C in pregnant women. Rev Esc Enferm USP.

[35] Diale Q, Pattinson R, Chokoe R, Masenyetse L, Mayaphi S (2015). Antenatal screening for hepatitis B virus in HIV-infected and uninfected pregnant women in the Tshwane district of South Africa. S Afr Med J.

[36] Murad EA, Babiker SM, Gasim GI, Rayis DA, Adam I (2013). Epidemiology of hepatitis B and hepatitis C virus infections in pregnant women in Sana'a, Yemen. BMC Pregnancy Childbirth.

[37] Jutavijittum P, Yousukh A, Saysanasongkham B, Samountry B, Samountry K, Toriyama K (2016). High rate of hepatitis B virus mother-to-child transmission in Lao people's Democratic Republic. Southeast Asian J Trop Med Public Health.

[38] Anaedobe CG, Fowotade A, Omoruyi CE, Bakare RA (2015). Prevalence, sociodemographic features and risk factors of hepatitis B virus infection among pregnant women in southwestern Nigeria. Pan Afr Med J.

[39] Noubiap JJ, Nansseu JR, Ndoula ST, Bigna JJ, Jingi AM, Fokom-Domgue J (2015). Prevalence, infectivity and correlates of hepatitis B virus infection among pregnant women in a rural district of the far north region of Cameroon. BMC Public Health.

[40] Souza MT, Pinho TL, Santos MD, Santos A, Monteiro VL, Fonsêca LM (2012). Prevalence of hepatitis B among pregnant women assisted at the public maternity hospitals of São Luís, Maranhão, Brazil. Braz J Infect Dis.

[41] Figueiredo NC, Page-Shafer K, Pereira FE, Miranda AE (2008). Serological markers for hepatitis B virus in young women attended by the family health program in Vitória, Espírito Santo, 2006. Rev Soc Bras Med Trop.

[42] Spano LC, Gatti J, Nascimento JP, Leite JP (2004). Prevalence of human cytomegalovirus infection in pregnant and non-pregnant women. J Inf Secur.

[43] Alvarado-Esquivel C, Hernández-Tinoco J, Sánchez-Anguiano LF, Ramos-Nevárez A, Cerrillo-Soto SM, Estrada-Martínez S (2014). Seroepidemiology of cytomegalovirus infection in pregnant women in Durango City, Mexico. BMC Infect Dis.

[44] Hamid KM, Onoja AB, Tofa UA, Garba KN (2014). Seroprevalence of cytomegalovirus among pregnant women attending Murtala Mohammed specialist hospital Kano. Nigeria Afr Health Sci.

[45] Inagaki AD, Oliveira LA, Oliveira MF, Santos RC, Araújo RM, Alves JA (2009). Seroprevalence of antibodies for toxoplasmosis, rubella, cytomegalovirus, syphilis and HIV among pregnant women in Sergipe. Rev Soc Bras Med Trop.

[46] Avila Moura A, de Mello MJ, Correia JB (2016). Serological statuses of pregnant women in an urban Brazilian population before and after the 2008 rubella immunization campaign. Vaccine.

